# Artificial neural network-based smart aerogel glazing in low-energy buildings: A state-of-the-art review

**DOI:** 10.1016/j.isci.2021.103420

**Published:** 2021-11-10

**Authors:** Yuekuan Zhou

**Affiliations:** 1 Sustainable Energy and Environment Thrust, Function Hub, The Hong Kong University of Science and Technology, Guangzhou, China; 2 Department of Mechanical and Aerospace Engineering, The Hong Kong University of Science and Technology, Clear Water Bay, Hong Kong SAR, China

**Keywords:** Artificial intelligence applications, Energy application, Surface treatment, Polymers

## Abstract

Aerogel materials with super-insulating, visual-penetrable, and sound-proof properties are promising in buildings, whereas the coupling effect of various parameters in complex porous aerogels proposes challenges for thermal/visual performance prediction. Traditional physics-based models face challenges such as modeling complexity, heavy computational load, and inadaptability for long-term validation (owing to boundary condition change, degradation of thermophysical properties, and so on). In this study, a holistic review is conducted on aerogel production, components prefabrication, modeling development, single-, and multi-objective optimizations. Methodologies to quantify parameter uncertainties are reviewed, including interface energy balance, Rosseland approximation and Monte Carlo method. Novel aerogel integrated glazing systems with synergistic functions are demonstrated. Originalities include an innovative modeling approach, enhanced computational efficiency, and user-friendly interface for non-professionals or multidisciplinary research. In addition, human knowledge-based machine learning can reduce abundant data requirement, increase performance prediction reliability, and improve model interpretability, so as to promote advanced aerogel materials in smart and energy-efficient buildings.

## Graphical abstract

**Figure:**
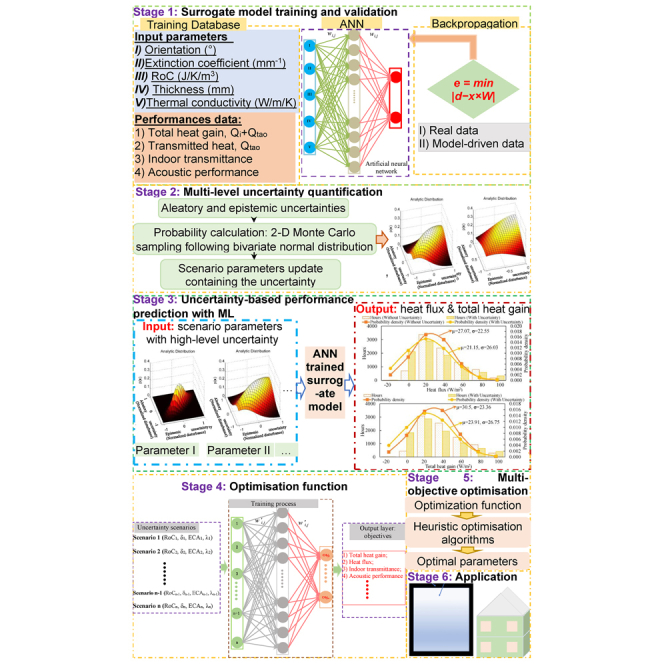

## Introduction

The daily increasing energy demands for accelerated economy development, social prosperity, and increased requirement on indoor environment, result in increased consumption of traditional fossil fuels and deteriorated environmental problems. The shortage of non-renewable energy (such as fossil fuels) will lead to energy crisis all over the world. Within the energy chain involving with energy supply, transmission, distribution, and energy consumptions of end-users, the building energy consumptions account for around more than 40% (Dincer, 2000). Furthermore, the 0.5 K urban overheating will increase the cooling load by around 1.84 kWh/m^2^ (Su et al., 2021). The realization of energy-efficient buildings with low energy consumption is one effective approach to replace the traditional fossil fuels, contributing to the carbon neutrality and mitigation on daily increased energy shortage crisis. It is noteworthy that energy consumptions, resulting from glazing systems (such as the penetrated solar radiation and the heat loss), account for around 40%–50% of the total building energy consumptions (Hee et al., 2015).

Over the past decades, researchers have focused on various operating solutions for the performance improvement of building glazing systems. The investigated solutions can be summarized as passive and active solutions (Jung et al., 2019). The passive solutions mainly include high emissivity surface (Xu and Raman, 2021), low-e glazing system, double-glazing system, air-vacuum layered triple glazed windows (Fang et al., 2020), reflective venetian blind (Wang and Chen, 2016), and naturally ventilated double skin façade (Wang et al., 2016). The active solutions mainly include the mechanically ventilated glazing systems (Cuce and Riffat, 2015). Compared to active operating strategies, passive solutions from the novel glazing system design with advanced materials are simpler and more flexible for realistic applications. The aerogel materials, as promising candidates, have attracted widespread interest recently, mainly owing to low-thermal conductivity, super-insulating, and sound-proof properties. The monolithic granular aerogels have been integrated with building glazing systems to reduce the heat loss in heating-dominated regions, such as Europe (Berardi, 2018; Talebi et al., 2019), and to reduce the total heat gain in cooling regions, such as subtropical Hong Kong (Zhou and Zheng, 2020a). In addition to aerogel glazing systems, the aerogels can also be integrated with building materials for energy savings, such as aerogel insulating panels (Yang et al., 2019a, 2019b), silica aerogel blankets (Nocentini et al., 2018), aerogel-based plaster (Ibrahim et al., 2018), aerogel-cement composites (Zeng et al., 2018), and aerogel-enhanced insulating materials (Yang et al., 2020).

Over the past several decades, aerogel glazings have been increasingly studied, from perspectives of thermo-physical parameters' identifications (such as extinction coefficient (Liu et al., 2019) and heat transfer coefficients (Tang et al., 2015), novel system designs (such as phase-change material-aerogel integrated window (Li et al., 2020b), and modeling development for multi-criteria performance predictions (thermal, optical and acoustic performances). Parametric analysis on aerogel glazing systems has been conducted to provide technical guidelines, in terms of thermal conductivity, thickness, and installation orientations. However, limited studies are on the novel structural designs, advanced modeling development, and stochastic uncertainty-based performance analysis for realistic operations. Furthermore, limited studies are on the advanced techniques for accurate performance predictions with uncertainty of thermo-physical parameters, uncertainty-based single- and multi-objective optimizations, and multi-criteria decision making. The systematic and comprehensive review is quite necessary to report the cutting-edge progress, together with potential challenges, outlooks, and recommendations for the promotion of upcoming research.

Aerogels' applications in buildings are reviewed, including aerogel production, component prefabrication, aerogel glazing system designs, together with robust optimal designs. Originalities include:

1) A holistic review of aerogel materials in buildings, from the perspectives of methodologies for critical parameters' identification, novel structural designs, mechanisms for heat transfer and light transmission, modeling development for multi-criteria predictions, and robust optimal designs.
2) Advanced machine learning methods in aerogel glazing systems, in terms of accurate predictions of multi-criteria performances (thermal, visual, and acoustic performances), scenario uncertainty-based system design, and optimizations with high computational efficiency and accuracy. Challenges are proposed to guide future research, including the development of advanced learning algorithms, the uncertainty quantification for multiple scenario parameters, and the heuristic optimization algorithms for global optimal solutions.
3) This review first presents the outlooks and recommendations for the promotion of aerogel materials in buildings, including advanced composite aerogel materials and novel phase-change materials (PCMs) integrated aerogel glazing systems, stochastic uncertainty-based optimizations, and economically competitive production of aerogel materials to promote the widespread acceptance in the market.

**Figure 1:**
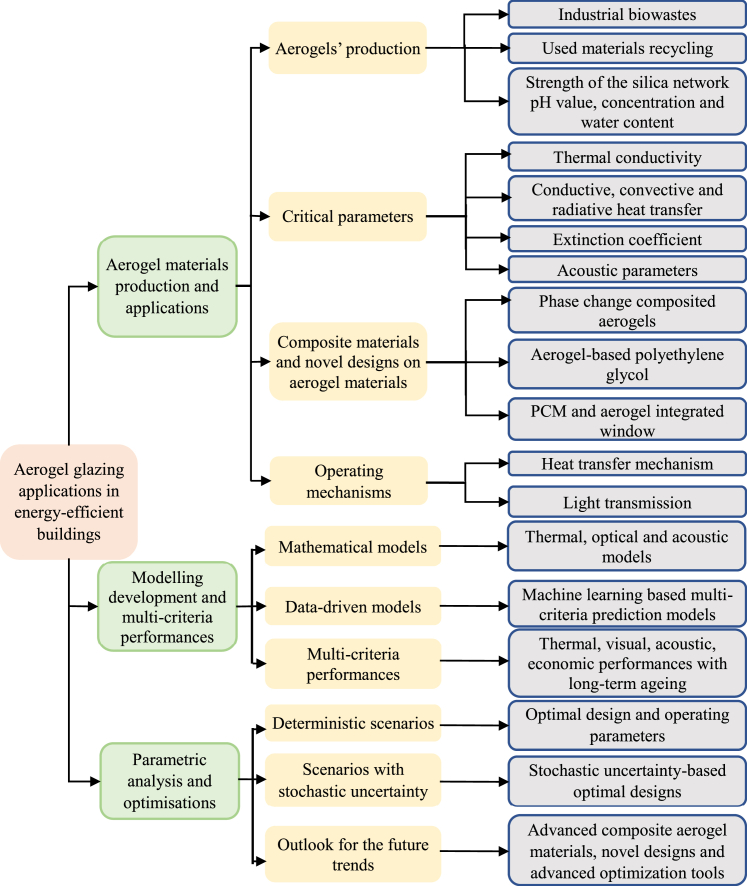
The roadmap for aerogel glazing applications in buildings components prefabrication, modeling development, and optimizations Aerogel glazing applications in energy-efficient buildings are reviewed from perspectives of aerogel materials production and applications, modeling development and multi-criteria performances, parametric analysis, and optimizations. The first Stage includes aerogels' production, identification of critical parameters, composite materials, and novel designs on aerogel materials and operating mechanisms. The modeling development for the multi-criteria predictions is in the second stage, including both mathematical and data-driven model. Considering the challenges for critical parameters' identification, such as the extinction coefficient, radiative thermal conductivity, and so on, the thermo-physical property-based mathematical model might be less reliable. Data-driven models are effective tools for thermal, visual, and acoustic performances predictions. Finally, approaches for multivariant and multi-objective optimizations with artificial neural network are proposed from the perspectives of deterministic scenarios and stochastic uncertainty.

Three main sections are included in this review, as demonstrated in Figure 1. The first section reviews aerogel production, component prefabrication, and building applications with five subsections, that is, aerogel production and thermo-physical properties, determination of critical parameters in aerogel materials, aerogels for novel glazing designs and advanced phase-change composited aerogels, mechanisms on heat transfer and light transmission, and aerogel glazing in different climate regions. In the second section, modeling development for multi-criteria predictions is systematically reviewed, including mathematical and data-driven models. In the third section, parametric analysis, single-, and multi-objective optimizations under deterministic scenario and stochastic uncertainty have been investigated. Outlooks and recommendations for aerogel materials' applications in buildings are demonstrated, including advanced composite aerogel materials and novel PCMs integrated aerogel glazing systems and stochastic uncertainty-based optimizations.

## Aerogel production, component prefabrication, and building applications

### Aerogel production and thermo-physical properties

Sources of aerogel production include industrial biowastes, such as rice husk (Li and Wang, 2008; Tang and Wang, 2005), bagasse (Nazriati et al., 2014), oil shale (Gao et al., 2010), fly (Shi et al., 2010), and wheat husk (Liu et al., 2015). Lignocellulosic biomasses, which are other sources of cellulose and lignin, have attracted increasing interest (Feng et al., 2015a, 2015b). Furthermore, researchers studied aerogel production by recycling used materials, such as banana peel, waste paper (Yue et al., 2018), waste newspaper (Jin et al., 2015), and waste cotton fabrics (Han et al., 2015).

In terms of aerogel production, the environmental impact between subcritical drying and supercritical aerogels was experimentally and comparatively analyzed (Pinto et al., 2020). Research results indicated that subcritical drying would cause lower environmental impact than supercritical aerogels. Furthermore, from the life cycle perspective, the production of raw materials consumes substantial energy. The pH value, concentration, and water content will affect the strength of the silica network (Smitha et al., 2006). For the regeneration purpose, the gel was washed with ethanol and heptane to completely remove the remaining water from the pores.

Aerogel fabrication was conducted for large-area deployment in buildings. Carbon-nanofiber aerogels from chemosynthesis and biosynthesis approaches are revised (Wu et al., 2018). Depending on the application components in buildings, aerogels are mainly applied in windows and envelopes. In terms of window, a nanoparticle-based mesoporous silica monolithic slab was produced for thermally insulated windows, with thermal conductivity of 0.104–0.160 W/(m·K) (Marszewski et al., 2019). With respect to the building envelope, foam concrete reinforced silica aerogel was applied in building envelopes with the thermal conductivity at 0.049 W/(m·K) (Liu et al., 2018). Sound absorption coefficient (SAC) of silica aerogel was studied in buildings (Talebi et al., 2019). They identified the characteristics of soundproof aerogel as lower bulk densities, larger pore size, and higher porosities. Moreover, the hydrophobic aerogel blanket generally shows higher SAC than the hydrophilic blankets. Waste tire fibers were recycled in aerogels for thermal insulation and sound absorption (Thai et al., 2020). This can provide frontier guidelines for large-scale and low-cost aerogel production and make good preparations for building applications.

Table 1 lists the properties of aerogel materials, including thermo-physical parameters, thermal insulation, acoustic, and structural properties. Thermal conductivity was quantified with the physical model (Zhang et al., 2017). The silica aerogel with the lowest thermal conductivity was 3.5 μm SiC particles. Studies on acoustic and structural properties indicated that the absorption coefficient can be improved from 0.1 to 0.29 for the aerogel-based plaster (Buratti et al., 2017), and the pressure-induced monolithic carbon aerogels show higher mechanical performance (Yang et al., 2019a, 2019b).

**Table 1:** A holistic overview on properties of aerogel materials

Research	Parameters	Results
Thermo-physical parameters	Thermal conductivity	1. Decrease of thermal conductivity from 24 to 13 mW/(m K) with granules compressed to a strain of 55–59% (or a bed density of 150–165 kg/m^3^) (Neugebauer et al., 2014).
		2. Thin aerogel equals to thick insulation materials (Guinoa et al., 2017).
	Extinction coefficient (Liu et al., 2019)	Solar extinction coefficient is dependent on meteorological conditions with a range between 0.0296 and 0.0392/mm.
	Heat loss and light transmittance	1. Thermal loss coefficient was lower than 0.7 W/m^2^K for a 15-mm aerogel (Schultz and Jensen, 2008).
		2. Compared to conventional double glazing, thermal loss in the aerogel glazing unit can be decreased by 63% when the particle size is lower than 0.5 mm (Gao et al., 2014).
Thermal insulation	Thermal insulation under various hygrothermal conditions (Lakatos, 2020)	A new methodology for thermal conductivity calculation of moist/frost insulation materials.
Acoustic parameters	Impact of granule size on acoustic performance (Buratti et al., 2017)	Compared to the traditional plaster, the absorption coefficient can be improved from 0.1 to 0.29 for the aerogel-based plaster.
Structural properties	Metal-organic framework-based monolithic carbon aerogel (Yang et al., 2019a, 2019b)	Pressure-induced monolithic carbon aerogels show higher mechanical performance and excellent energy storage capacity.

The properties of aerogel materials are summarized, including thermo-physical parameters, thermal insulation, acoustic, and structural properties. Thermal conductivity was quantified with the physical model (Zhang et al., 2017). The silica aerogel with the lowest thermal conductivity was 3.5 μm SiC particles. Studies on acoustic and structural properties indicated that the absorption coefficient can be improved from 0.1 to 0.29 for the aerogel-based plaster (Buratti et al., 2017), and the pressure-induced monolithic carbon aerogels show higher mechanical performance (Yang et al., 2019a, 2019b).

### Determination of critical parameters in aerogel materials

Before the real application of aerogel materials, the accurate identification of critical parameters is critical for thermal and energy performance analysis. Studies are mainly on the development of accurate methodologies for accurate identifications of extinction coefficient, radiation, and convection in porous aerogel materials. Furthermore, VO2-aerogel hybrid film was prepared with adjustable transmissivity for window retrofits. An optically switchable and thermally insulating VO2-aerogel hybrid film was developed (Zhao et al., 2020) with a U-value of around 3.0 W/(m^2^·K), the luminous transmittance higher than 60%, and a solar modulation ability of around 20%.

#### Extinction coefficient

The spectral and Rosseland mean extinction coefficients were quantified (Zhang et al., 2018), with the elimination of traditional assumptions. Without the consideration of practical absorption and surface reflection, the measured mean extinction coefficient is overestimated by about 15%–18%, and by about 18%–33%, respectively.

In order to quantify the solar extinction coefficient, the interface energy balance was adopted (Liu et al., 2019). Research results showed that the solar extinction coefficient is dependent on meteorological conditions. For clear and overcast sky conditions, the solar extinction coefficient is 0.0392/mm and 0.0296/mm. The global extinction coefficient is independent of the gas pressure (Liu et al., 2017).

The temperature-averaged extinction coefficient (β) for wavelength range between λ_1_ and λ_2_ is shown later in discussion (Yu et al., 2014):

(Equation 1) $$\beta \left(T\right)={\left[\int_{0}^{\infty }\frac{1}{\beta_{\lambda }}\frac{\partial E_{b\lambda }\left(T\right)}{\partial E_{b}\left(T\right)}d\lambda \right]}^{-1}=\frac{\int_{\lambda_{1}}^{\lambda_{2}}E_{b\lambda }\left(T\right)d\lambda }{\int_{\lambda_{1}}^{\lambda_{2}}\frac{1}{\beta_{\lambda }}\frac{\partial E_{b\lambda }\left(T\right)}{\partial E_{b}\left(T\right)}d\lambda }$$

#### Heat transfer in porous aerogels

For the heat transfer in porous aerogels, studies are on the coefficients of conductive, convective, and radiative heat transfer. In accordance with the characteristic of radiative heat transfer, the radiative heat transfer increases with the temperature rise, but decreases with the gas pressure rise. Furthermore, the constitution of heat transfer in the porous aerogels is highly dependent on the temperature. They indicated that when the temperature is lower than 600 K, the main heat transfer source is conduction (more than 93%) (Liu et al., 2017).

Methodologies for the accurate identification of heat transfer in porous aerogels have been investigated in academia. Rosseland approximation was adopted to estimate the thermal conductivity and the radiative conductivity of aerogel materials (Pierre et al., 2017). The radiative conductivity can be useful for a mean extinction coefficient through the Rosseland approximation. The T-matrix algorithm was adopted to calculate the radiative properties of aerogels (Yu et al., 2014). Parametrical analysis results indicated that radiative properties are independent of the nanoparticle sizes. In addition, the stochastic uncertainty method has been adopted to estimate the radiative thermal conductivity. Extinction coefficient was modified using the Monte Carlo to accurately predict the radiative thermal conductivity (Zhao et al., 2015). Compared with existing methods, the modified model can avoid the underestimation of the mean temperature.

The radiation results from the electromagnetic radiation emitted by all surfaces with temperature difference, and the radiative conductivity is shown later in discussion (Tang et al., 2015):

(Equation 2) $$\lambda_{r}=\frac{16\sigma n^{2}T^{3}}{3K_{e,m}}$$

where n is the materials' effective refractive index, σ is the Stefan-Boltzmann constant, T is the mean temperature, and $K_{e,m}$ is the Rosseland mean extinction coefficient.

(Equation 3) $$\frac{1}{K_{e,m}}=\frac{\int_{0}^{\infty }\frac{1}{k_{\lambda }}\frac{\partial e_{b\lambda }}{\partial \left(T\right)}d\lambda }{\int_{0}^{\infty }\frac{\partial e_{b}}{\partial \left(T\right)}d\lambda }=\int_{0}^{\infty }\frac{1}{k_{\lambda }}\frac{\partial e_{b\lambda }}{\partial e_{b}}d\lambda$$

where $e_{b\lambda }$ is the spectral hemispherical blackbody flux and $e_{b}$ is the hemispherical blackbody flux.

For an optically thick medium, an effective radiative conductivity, *k*_*r*_*(T)* is calculated later in discussion:

(Equation 4) $$k_{r}\left(T\right)=\frac{16\sigma_{b}T^{3}}{3\beta \left(T\right)}$$

### Aerogels for novel glazing designs and advanced phase-change composited aerogels

Regarding the application of aerogel materials in building systems, one of the most common applications is thermal insulation, with the exploitation of low thermal conductivity on aerogel materials. A holistic overview of aerogel-based super-insulating components in buildings was provided (Cuce et al., 2014a, 2014b). The state-of-the-art review presents promising prospects of aerogels for energy savings in the building. The monolithic granular aerogel was used in glazing systems owing to the super insulation (Baetens et al., 2011) and high absorption (Lallich et al., 2009).

**Figure 2:**
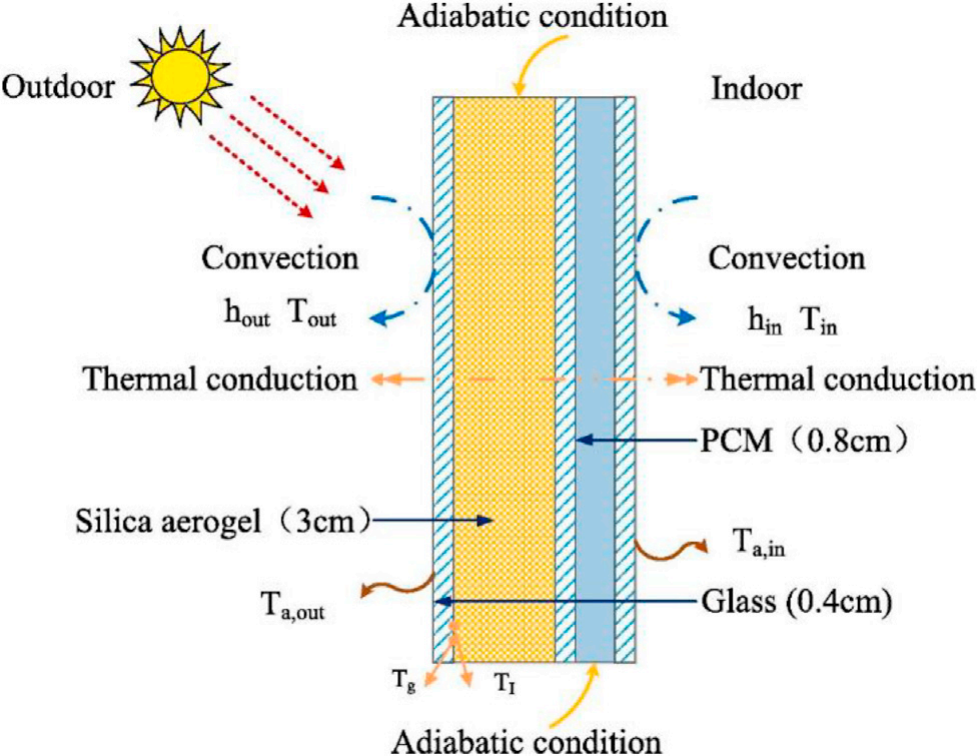
Structural configuration of a PCM and aerogel integrated window glazing system The structural configuration of a PCM integrated aerogel glazing system includes silica aerogel, PCM, and glass layers. The exterior aerogel layer can provide super-insulation with a relatively low thermal conductivity at 0.018 W/(m K). The interior PCM is charged by the daytime solar radiation, and then the stored thermal energy is discharged to maintain the indoor thermal comfort during the nighttime. An aerogel-PCM glass window was designed for heating applications (Li et al., 2020b). Figure 2 is reprinted from, Applied Thermal Engineering. Li et al. (2020b). Thermal performance evaluation of glass window combining silica aerogels and phase-change materials for the cold climate of China. Copyright with permission from Elsevier

The synergistic functions between aerogels and other materials have been exploited, through the novel structural designs. Figure 2 demonstrates the structural configuration of a PCM integrated aerogel glazing system. The underlying mechanism is that the exterior aerogel layer can provide super-insulation with a relatively low thermal conductivity at 0.018 W/(m K). The interior PCM is charged by the daytime solar radiation, and then the stored thermal energy is discharged to maintain the indoor thermal comfort during the nighttime. An aerogel-PCM glass window was designed for heating applications (Li et al., 2020b). Thermal conductivity and thickness dominate the thermal performance, and the silica aerogel can exploit the PCM latent heat (Li et al., 2020b). An experimental study on the thermal and optical performance of a passive solar wall, consisting of silica aerogel and PCM, showed that the passive wall can keep the indoor air temperature 9°C higher than outside air and provide up to 500 lux to indoor environment (Berthou et al., 2015). The optimization on the geometrical and thermophysical parameters of PCM and aerogel-filled multiple glazing indicates that the energy-saving performance is highly dependent on the optical properties of glass, in the condition that the radiation is above 2.5 μm (Zhang et al., 2021). A comprehensive review was conducted on the optical and thermal performance of PCM glazing for thermal energy storage and solar transmission (Li et al., 2020b).

In addition, the aerogels have been integrated into PCMs, foam concrete, polyethylene glycol to produce advanced composite materials. Phase-change composited aerogels (PCCA) are designed for thermal insulation and temperature control (Li et al., 2020c). The additives of 40% phase-change microcapsules in the composite can achieve the enthalpy and fusion temperature at about 25.0°C and 52.78 J/g. A new PCM with graphene aerogel was produced and applied in the encapsulated polyethylene glycol (Liao et al., 2020). Compared to the polyethylene glycol, the composite PCM shows higher latent heat, higher thermal conductivity, and better shape stability, for solar energy harvesting, energy storage, and management. An MXene aerogel-based polyethylene glycol was produced (Lin et al., 2020) for solar energy utilization. Advantages for the composite PCM material include the stability during phase-change process through the MXene skeleton and improved photothermal storage efficiency.

### Mechanisms on light transmission and heat transfer

Owing to the sophisticated nano-porous structure of aerogels, conduction, convection, and radiation simultaneously appear. The uncertainties of several critical parameters (such as extinction coefficient, radiative conductivity, reflection coefficient, and so on) make the modeling process more complex.

**Figure 3:**
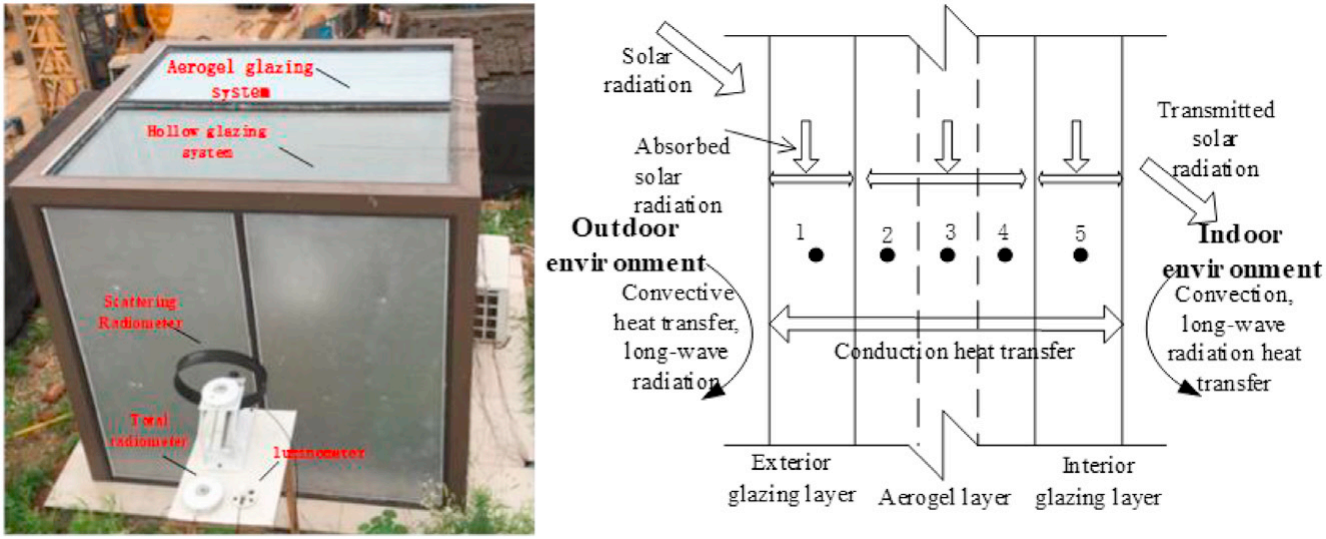
Aerogel glazing in buildings and schematic diagram. (A) On-site platform (Chen et al., 2018): there are two testing thermal zones, that is, one is the reference case with hollow glass, and one is the experimental case with aerogel glazing. The super-insulating aerogel material was uniformly distributed in the middle of a double-glazing system. (B) Schematic diagram of an aerogel glazing system. The finite-difference method is adopted to calculate the dynamic thermal and optical performance. Heat transfer processes in the aerogel layer include conduction, convection, and radiation. Heat transfer processes in the aerogel glazing system include heat conduction in each solid glass layer, natural convection, and long-wave radiation between exterior/interior surfaces and surroundings. Figure 3 is reprinted from, Energy, Chen et al., (2018). Dynamic heat transfer model and applicability evaluation of aerogel glazing system in various climates of China. Copyright 163, 1115–1124. with permission from Elsevier.

Figure 3A shows the application of an aerogel glazing system in a testing chamber. In this section, the mechanisms of heat transfer and light transmission are demonstrated through an already developed integrated model, by the author (Zhou and Zheng, 2020a). Depending on the incident angle of solar radiation and the extinction coefficient, the solar radiation will be partially absorbed by the aerogel and then it will be converted into thermal energy, as demonstrated in Figure 3B. Depending on the thermal conductivity, the accumulated heat discharging rate (or called heat flux) will dynamically affect the indoor cooling load. In addition, the solar radiation will partially penetrate the aerogel layer, becoming the transmitted heat source. Table 2 lists the integrated thermal and optical models. In order to develop the accurate optimal model, several parameters need to be identified, including the transmittance of each layer, absorption coefficient of incident radiation, and so on.

**Table 2:** Integrated thermal and optical models of the aerogel glazing system

	Layers	Heat transfer model &&indoor illuminance	Supplementary
The heat transfer model	Exterior glazing layer	$\rho_{1}C_{p1}\delta_{1}\frac{T_{1}^{k+1}-T_{1}^{k}}{\Delta \tau }=\;h_{ce}\left(T_{e}^{k}-T_{1}^{k}\right)+h_{re}\left(T_{e}^{k}-T_{1}^{k}\right)+k_{1}\left(T_{2}^{k}-T_{1}^{k}\right)+\alpha_{1}I_{0}$ (5)	$k_{1}=\frac{2\cdot \lambda_{1}\cdot \lambda_{2}}{\lambda_{1}\cdot \lambda_{2}/3+\lambda_{2}\cdot \delta_{1}}$;$k_{2}=\frac{\lambda_{2}}{\delta_{1}/3}$ $h_{ra}=\sigma \varepsilon_{5}\left(T_{a}^{k^{2}}+T_{5}^{k^{2}}\right)\;\left(T_{a}^{k}+T_{5}^{k}\right)$ $h_{re}=\sigma \varepsilon_{1}\left(T_{sky}^{k^{2}}+T_{1}^{k^{2}}\right)\left(T_{sky}^{k}+T_{1}^{k}\right)\left(T_{sky}^{k}-T_{1}^{k}\right)/\left(T_{e}^{k}-T_{1}^{k}\right)$ $h_{ca}=5.34+3.27\nu_{a}$;$h_{ce}=5.62+3.9\nu_{e}$ $T_{sky}^{k}$ = 0.0552${\left(T_{amb}^{k}\right)}^{1.5}$
	Aerogel layer	$\rho_{2}C_{p2}\frac{\delta_{2}}{3}\frac{T_{2}^{k+1}-T_{2}^{k}}{\Delta \tau }=\;k_{1}\left(T_{1}^{k}-T_{2}^{k}\right)+k_{2}\left(T_{3}^{k}-T_{2}^{k}\right)+\frac{1}{3}\alpha_{2}I_{0}$ (6)	
		$\rho_{2}C_{p2}\frac{\delta_{2}}{3}\frac{T_{3}^{k+1}-T_{3}^{k}}{\Delta \tau }=\;k_{2}\left(T_{2}^{k}-T_{3}^{k}\right)+k_{2}\left(T_{4}^{k}-T_{3}^{k}\right)+\frac{1}{3}\alpha_{2}I_{0}$ (7)	
		$\rho_{2}C_{p2}\frac{\delta_{2}}{3}\frac{T_{4}^{k+1}-T_{4}^{k}}{\Delta \tau }=\;k_{2}\left(T_{3}^{k}-T_{4}^{k}\right)+k_{1}\left(T_{5}^{k}-T_{4}^{k}\right)+\frac{1}{3}\alpha_{2}I_{0}$ (8)	
	Interior glazing layer	$\rho_{1}C_{p1}\delta_{1}\frac{T_{5}^{k+1}-T_{5}^{k}}{\Delta \tau }=\;h_{ca}\left(T_{a}^{k}-T_{5}^{k}\right)+h_{ra}\left(T_{a}^{k}-T_{5}^{k}\right)+k_{1}\left(T_{4}^{k}-T_{5}^{k}\right)+\alpha_{5}I_{0}$ (9)	
	Indoor air	${\left(\rho c\right)}_{a}\frac{T_{a}^{k+1}-T_{a}^{k}}{\Delta \tau }=h_{ca}\left(T_{5}^{k}-T_{a}^{k}\right)+\sigma \varepsilon_{5}\left(T_{5}^{k^{4}}-T_{a}^{k^{4}}\right)\;$ (10)	
The optical model	Absorption coefficient of incident radiation	$\alpha_{K\to K+1}=\varphi_{K}\left(1-\tau_{K\to K+1}+\frac{1-\tau_{K\to K+1}}{\beta_{K}\tau_{K\to K+1}}\right)\prod_{i=1}^{K-1}\left(\varphi_{i}\tau_{i\to i+1}\right)$ (11)	
	Transmittance	$\tau =\prod_{K=1}^{4}\left(\varphi_{K}\tau_{K\to K+1}\right)$ (12)	
	Equivalent reflectivity of each interface	$\left\{ \begin{array}{l} \beta_{K}^{\prime }=\beta_{K}\tau_{K\to K+1}^{2}\\ \beta_{K}=1-\varphi_{K}\left(1-\beta_{K}^{\prime }\right) \end{array}\right.$ (13)	
Diffuse illuminance	South	*E*_*dv,S*_*= −1.9541+1.3762×E*_*dh,S*_*−0.00152×E*^*2*^_*dh,S*_ (14)	$\text{Edv}\left(\alpha \right)=\left\{ \begin{array}{l} \begin{array}{l} \frac{\alpha }{\left(90{}^{\circ}-0{}^{\circ}\right)}\times E_{dv,S}+\frac{\left(90{}^{\circ}-\alpha \right)}{\left(90{}^{\circ}-0º\right)}\times E_{dv,W}\;0{}^{\circ}\leq \alpha <90{}^{\circ}\;\\ \frac{\alpha -90{}^{\circ}}{\left(180{}^{\circ}-90{}^{\circ}\right)}\times E_{dv,W}+\frac{\left(180{}^{\circ}-\alpha \right)}{\left(180{}^{\circ}-90{}^{\circ}\right)}\times E_{dv,N}\;90{}^{\circ}\leq \alpha <180{}^{\circ} \end{array}\\ \begin{array}{l} \frac{\alpha -180{}^{\circ}}{\left(270{}^{\circ}-180{}^{\circ}\right)}\times E_{dv,N}+\frac{\left(270{}^{\circ}-\alpha \right)}{\left(270{}^{\circ}-180{}^{\circ}\right)}\times E_{dv,E}\;180{}^{\circ}\leq \alpha <270{}^{\circ}\\ \frac{\alpha -270{}^{\circ}}{\left(360{}^{\circ}-270{}^{\circ}\right)}\times E_{dv,E}+\frac{\left(360{}^{\circ}-\alpha \right)}{\left(360{}^{\circ}-270{}^{\circ}\right)}\times E_{dv,S}\;270{}^{\circ}\leq \alpha <360{}^{\circ} \end{array} \end{array}\right.$
	East	*E*_*dv,E*_*=0.4205+1.5211×E*_*dh,E*_*−0.0182×E*^*2*^_*dh,E*_ (15)	
	West	*E*_*dv,W*_*=−1.795+0.9643×E*_*dh,W*_*−0.0082×E*^*2*^_*dh,W*_ (16)	
	North	*E*_*dv,N*_*= −0.9582+0.9045×E*_*dh,N*_*−0.0072×E*^*2*^_*dh,N*_ (17)	
Direct illuminance	South	*E'*_*dv,S*_*= E*_*dn,S*_*×cos(β*_*S*_*)* (18)	$E^{\prime }dv\left(\alpha \right)=\left\{ \begin{array}{l} \begin{array}{l} \frac{\alpha }{\left(90{}^{\circ}-0{}^{\circ}\right)}\times E\prime_{dv,S}+\frac{\left(90{}^{\circ}-\alpha \right)}{\left(90{}^{\circ}-0{}^{\circ}\right)}\times E\prime_{dv,W}\;0{}^{\circ}\leq \alpha <90{}^{\circ}\;\\ \frac{\alpha -90{}^{\circ}}{\left(180{}^{\circ}-90{}^{\circ}\right)}\times E\prime_{dv,W}+\frac{\left(180{}^{\circ}-\alpha \right)}{\left(180{}^{\circ}-90{}^{\circ}\right)}\times E\prime_{dv,N}\;90{}^{\circ}\leq \alpha <180{}^{\circ} \end{array}\\ \begin{array}{l} \frac{\alpha -180{}^{\circ}}{\left(270{}^{\circ}-180{}^{\circ}\right)}\times E\prime_{dv,N}+\frac{\left(270{}^{\circ}-\alpha \right)}{\left(270{}^{\circ}-180{}^{\circ}\right)}\times E\prime_{dv,E}\;180{}^{\circ}\leq \alpha <270{}^{\circ}\\ \frac{\alpha -270{}^{\circ}}{\left(360{}^{\circ}-270{}^{\circ}\right)}\times E\prime_{dv,E}+\frac{\left(360{}^{\circ}-\alpha \right)}{\left(360{}^{\circ}-270{}^{\circ}\right)}\times E\prime_{dv,S}\;270{}^{\circ}\leq \alpha <360{}^{\circ} \end{array} \end{array}\right.$
	East	*E'*_*dv,E*_*= E*_*dn,E*_*×cos(β*_*E*_*)* (19)	
	West	*E'*_*dv,W*_*= E*_*dn,W*_*×cos(β*_*W*_*)* (20)	
	North	*E'*_*dv,N*_*= E*_*dn,N*_*×cos(β*_*N*_*)* (21)	

where *E* denotes the diffuse illuminance; α is the orientation angle of the surface; *E′* denotes the direct normal illuminance; *β* denotes the incident angles. *K*_*g*_ and *K*_*a*_ refer to extinction coefficients of glazing and aerogel layers.

Table 2 is reprinted from the Journal of Cleaner Production, Zhou and Zheng (2020a). Machine learning-based multi-objective optimization of an aerogel glazing system using NSGA-II—study of modeling and application in the subtropical climate Hong Kong. Copyright 253, 119,964, with permission from Elsevier.

## Modeling development and multi-criteria assessment

Over the past several decades, researchers are mainly focused on accurate predictions of thermal, optical, and energy performances. The performance prediction tools can be classified into mathematical models and data-driven models. The assessment criteria of aerogel glazing systems include thermal performances (such as total heat gain and thermal loss), visual performance (indoor illuminance), and acoustic performance.

### Mathematical models

**Figure 4:**
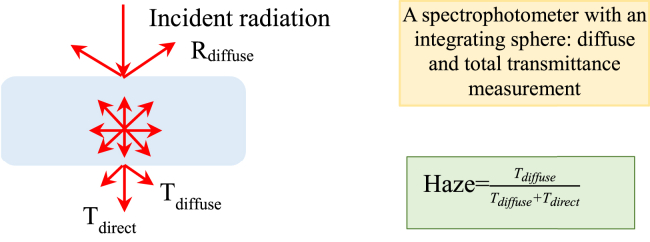
Experimental database preparation for haze calculation A spectrophotometer with an integrating sphere can measure diffuse and total transmittance of aerogel materials with respect to different samples, aerogel density, mean particle radius, wavelength, and etc. Afterward, the haze can be mathematically calculated.

Table 3 lists a holistic overview on mathematical models of aerogel glazing systems, including heat transfer models and optical models. Through the on-site experimental calibration, a heat transfer model using the classical heat conduction differential equations was developed (Zheng and Zhou, 2019). The model can realize the maximum absolute error at 3.4°C between the experiment and simulation. A model was developed to predict daylight illuminance (Garnier et al., 2015). Compared with other visual transmission models, the experimentally fitted visual transmission equation shows higher accuracy in predicting daylight illuminance. In terms of the haze, Figure 4 shows the experimental testing and mathematical calculation. As shown in Figure 4, the haze is the ratio of diffuse radiation (T_diffuse_) to the sum of diffuse and direct radiation (T_diffuse_ + T_direct_).

**Table 3:** Mathematical model development of aerogel glazing systems

Modeling type	Studies	Systems	Methodology	Results
Heat transfer model	Zheng and Zhou (2019)	Double glazing with aerogel material	Classical heat conduction differential equation	Absolute error at 3.4°C was between experiment and simulation.
	Li et al. (2020a, 2020b, 2020c)	PCMs integrated aerogel glazing	Parametrical analysis based on a validated model	The PCM can be completely charged/discharged when the thickness of silica aerogel is 30 mm.
Optical model	Zheng and Zhou (2019)	Double glazing with aerogel material	Interface energy balance method	The absolute error is 20 W/m^2^.
	Liu et al. (2019)	Double glazing with aerogel material	Extinction coefficient estimation through an optical model	Solar extinction coefficient is dependent on weather condition: the extinction coefficient is 0.0392/mm and 0.0296/mm for clear and overcast sky, respectively.
	Garnier et al. (2015)	Super-insulated aerogel windows	Daylight illuminance model	Compared with other visual transmission equations, the experimentally fitted visual transmission equation shows more accuracy in daylight illuminance prediction.

The models include heat transfer models and optical models.

Based on the review of mathematical models, disadvantages can be generally summarized later in discussion:

1) physics-based models have challenges to accurately predict the complicated thermal and visual performances of aerogel glazing systems, especially considering degradation in thermophysical properties over a long time and boundary condition change (such as meteorological parameters). Based on our experimental and numerical study, although the 2-day validation between the physical model and experimental data is quite good, the model will be less robust if we conduct the long-term validation.
2) physics-based model development is complex to accurately characterize the dynamic heat transfer, solar radiation transmission, and indoor illuminance of an aerogel glazing system. Normally, hybrid models are required in the physics-based approach, such as optical model and heat transfer model. Furthermore, these models need to interact with each other instead of separation, to characterize the coupling effect of many parameters in complex porous aerogels (such as solar radiation transmission, as shown in our previous work (Zheng and Zhou, 2019)), increasing the difficulty, and complexity for accurate model development.
3) high computational load is required owing to the convergence through iterative calculation. Furthermore, the computational load will be extremely high when parametrical analysis, comparative analysis, and optimal design are required.

### Data-driven models

#### Aerogel glazing applications in different climate regions

Table 4 summarizes the aerogel glazing applications in different climatic zones. As listed in Table 4, the aerogel glazing is technically feasible in cold regions (Chen et al., 2018). The energy-saving magnitude of aerogel glazing is highly dependent on local climate conditions (Zhou and Zheng, 2020b).

**Table 4:** Summary of aerogel glazing applications in different climates

Studies	Climate zones	Systems	Building types	Methodology	Research results
Huang and Niu (2015)	Subtropical climates	aerogel glazing system in building façade	High-rise commercial building	Numerical simulation with experimentally tested thermal and optical parameters	Decrease of the cooling load by 60% and energy consumption by 4%
Zhou and Zheng (2020a)	Hot summer and warm winter	aerogel glazing system	–	Multi-objective optimization	The biobjective optimizations can reduce the total heat gain, but the annual indoor illuminance.
Zhou and Zheng (2020c)	Hot summer and warm winter	aerogel glazing system	–	Stochastic uncertainty analysis	The scenario uncertainty will decrease the heat flux and total heat gain.
Zhou and Zheng (2020d)	Subtropical regions	aerogel glazing system	–	Stochastic uncertainty-based optimization	The uncertainty-based optimization can reduce the heat flux by 31.5%, and the total heat gain by 28.3%.
Cotana et al. (2014)	Cold climates	aerogel glazing in the south facade	Experimental testing chamber	Comparative analysis between aerogel and air-filled glazing, in terms of thermal-energy, lighting, and acoustic performances	Energy consumption can be reduced by 50%.
Berardi (2015)	Different climates	monolithic aerogel window	Education and office buildings	Thermal and lighting performances in various climates	Aerogel glazing systems are energy-saving in different climates.
Chen et al. (2018)	Different climates	aerogel glazing system	–	Dynamic model development	Aerogel glazing system is technically feasible in cold regions, whereas the feasibility of the system is dependent on the installed position in hot summer and cold winter regions.
Zhou and Zheng (2020b)	Different climates	aerogel glazing system	–	Climate adaptive optimal design	The heat gain can be reduced by 62.5% in cold regions, and by 5.9% in subtropical regions.

Climate zones mainly include cooling climates, hot summer and warm winter, subtropical regions, heating climates. Building types mainly include commercial, education, and office buildings.

In the subtropical region, the cooling load can be reduced by 60% (Huang and Niu, 2015). In addition to the parametrical analysis, solutions for the performance enhancement include scenario uncertainty analysis, deterministic and uncertainty-based optimization, and multi-objective optimization. Based on the study in a subtropical region, Guangzhou, scenario uncertainty, and uncertainty-based optimization will reduce the annual heat flux from 237.2 to 185.3 kWh/(m^2^.a) by 21.9% (Zhou and Zheng, 2020c) and from 237.2 to 162.54 kWh/m^2^.a by 31.5% (Zhou and Zheng, 2020d), respectively. Furthermore, when considering the contradiction of multi-objectives, the biobjective optimizations in Hong Kong can reduce the total heat gain but increase the indoor illuminance. In addition to subtropical regions, thermal and energy performances in cold regions have also been studied (Berardi, 2018; Gao et al., 2016a, 2016b) for thermal insulation and energy saving.

In cold climate regions, transparent aerogel was applied in glazing systems for solar energy harvesting. The transparent aerogel was applied in glazing systems with high solar transmittance at 65%, together with heat insulation performance and heat transfer coefficient of 0.4 W/(m^2^·K) (Reim et al., 2002). Energy performance of aerogel window in cold climate regions was analyzed (Buratti and Moretti., 2012a, 2012b). Results showed that compared to traditional windows, the 4-mm monolithic aerogel glass can reduce heat losses by 62%. The solar transmittance was 53% and 88% for a 10-mm silica aerogel semi-translucent sphere and a highly translucent granulate, respectively (Reim et al., 2005). In the cold climate of China, latent heat storage material was integrated into a silica aerogel window for solar energy storage and thermal insulation (Li et al., 2020b).

#### Justification and mechanisms on data-driven models

Professional knowledge is required for physics-based models, such as conductive, convective, and radiative heat transfer mechanisms within a porous nano-particle structure. Aleatory and epistemic uncertainties of thermo-physical properties in porous aerogels will make accurate predictions more challenging. To simplify the prediction of thermal, visual, and energy performances, machine learning techniques can be employed to learn underlying mechanisms of heat transfer and light transmission, according to the experimentally testing results. The underlying mechanism is to implement an error-driven rule to update the learning matrix, with the objective to minimize the prediction error. The back-propagation algorithm (Rumelhart et al., 1986) is adopted to quantify errors of hidden nodes. In the current academia, applications of supervised machine learning method in aerogel glazing were studied, in terms of predictions on thermal and energy performances (Zheng and Zhou, 2019), indoor illuminance (Zhou and Zheng, 2020a), and uncertainty-based analysis (Zhou and Zheng, 2020c).

An artificial neural network-based model was developed to predict the heat flux and the total heat gain (Zheng and Zhou, 2019). The model can realize accurate predictions on thermal performances. With respect to the indoor illuminance, the diffuse, and direct illuminance of each aerogel glazing at different orientations were calculated (Zhou and Zheng, 2020a), based on the empirical equations in the literature. Research results indicate that in cooling-dominated regions, the indoor illuminance will contradict thermal performances.

**Figure 5:**
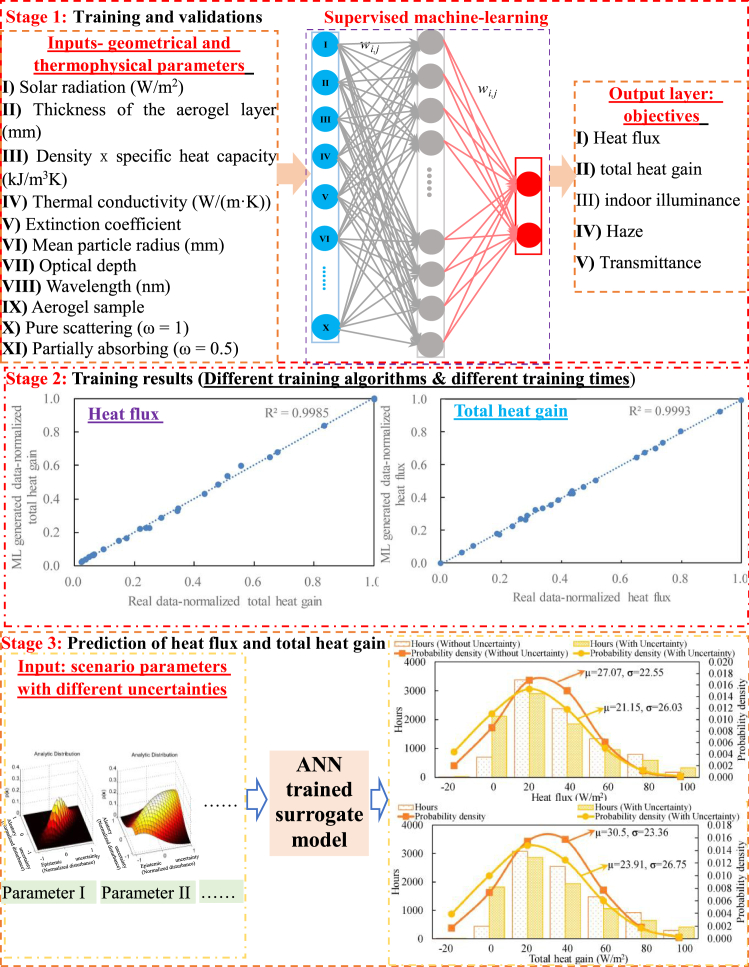
Model development: training process and multi-criteria performance predictions In Stage 1, supervised machine learning is adopted to train the surrogate model, with the straightforward mathematical association between multi-variables and multi-objectives. In Stage 2, the training results on different objectives (i.e., heat flux and total heat gain) are shown, with respect to different training algorithms (stochastic gradient descent function, batch function, the sum of square error function, and the cross-entropy function) and different training times. In terms of total heat gain, the coefficient of determination (R2) is 0.9985 and 0.9993 when the training time is 5000 and 30,000, respectively. In terms of heat flux, the coefficient of determination (R2) is 0.9993 and 0.9997 when the training time is 5000 and 30,000, respectively. In Stage 3, based on the developed surrogate model, the heat flux and total heat gain are predicted for the case with different uncertainty levels of input parameters (Zhou and Zheng, 2020c). Figure 5 is partially reprinted from, Energy, Zhou and Zheng (2020c). Uncertainty study on thermal and energy performances of a deterministic parameters-based optimal aerogel glazing system using the machine-learning method. Copyright 193, 116,718. with permission from Elsevier

Figure 5 demonstrates the approaches for the data-driven model to predict multi-criteria performances. Stage 1 includes both training and validation processes. The training database includes input parameters (e.g., geometrical and thermophysical parameters) and multi-criteria performances (e.g., heat gain, indoor illuminance, haze, and so on). The training database is mainly established from experimental testing results. However, considering the labor cost for experiments and the time-consuming of real-time experimental data preparation, the training database can also be established by experimentally calibrated numerical models. The training process is to dynamically update the weighting matrix, through the backpropagated errors between learning results and the real data. The performance evaluation of the predicted results is conducted in Stage 2, with respect to different structural configurations of neural networks (such as different setting numbers of hidden layers), different training algorithms, and different training epochs. Afterward, multi-criteria performances are predicted in Stage 3, using the well-trained data-driven models.

Compared to the mathematical model, the main advantages of the data-driven models include no requirement on professional knowledge of heat transfer and light transmission, and the computational-efficient performance prediction. Justifications on the necessity of data-driven models are summarized later in discussion:

1) through the feature extraction and classification, data-driven models can actively adapt to the complicated environment (such as variation of meteorological parameters, boundary condition change, degradation on thermophysical properties, and so on) and make relatively accurate predictions based on the multiple linear regression, the support vector regression, and the backpropagation neural network.
2) the data-driven model can provide a user-friendly interface for non-professionals or multidisciplinary research works and address the coupling effects of many parameters in complex porous aerogels.
3) interdisciplinary machine learning techniques and advanced algorithms can assist the robust design for stochastic uncertainty and uncertainty-based optimization, to achieve climate-adaptive aerogel glazing systems with superior thermal/visual/acoustic performances.

However, in order to guarantee the performance prediction accuracy of the well-trained surrogate model via machine learning, a large amount of the database needs to be prepared. Furthermore, the prediction accuracy relies on the learning algorithms, structural configuration of the neural network, and the total learning epochs. Depending on the complexity of aerogel integrated systems, researchers can develop accurate models for multi-criteria performance predictions, from perspectives of different learning algorithms, different structural configurations, and together with different learning epochs.

### Multi-criteria on aerogel glazing systems

In academia, multi-criteria performances have been studied, for the widespread aerogel applications, including building energy savings in different climates, indoor illuminance, acoustic performance, economic performance, and so on, as listed in Table 5.

**Table 5:** A summary of the multi-criteria performances of aerogel-based systems

Multi-criteria performances	Studies	Systems	Conclusions
Building energy savings			
Cooling climates	Huang and Niu (2015)	Aerogel glazing system in building façade	Decrease of cooling load by 60%, and energy consumption by 4%.
Heating climates	Cotana et al. (2014)	Aerogel glazing in the south facade	Energy consumption can be reduced.
Different climates	Berardi (2015)	Monolithic aerogel window	Aerogel glazing systems are energy-saving.
Indoor illuminance	Moretti et al. (2018)	Polycarbonate with granular aerogel	Light transmittance can be decreased.
	Cotana et al. (2014)	Aerogel glazing in the south facade	Daily illuminance can be reduced during sunny days, and indoor illuminance can be increased on a cloudy day.
	Garnier et al. (2015)	Aerogel glazing system	Aerogel glazing can improve diffuse natural light.
	Zinzi et al. (2019)	Granular and monolithic aerogel glazing	The light transmission of the monolithic silica aerogel glazing is 10% higher than a granular aerogel glazing, but 13% lower than conventional low-e double glazing
Acoustic performance	Cotana et al. (2014)	Aerogel glazing in the south façade	The aerogel can increase the acoustic insulation level from 28 to 31 dB.
	Ibrahim et al. (2019)	Aerogel insulation wall	The aerogel insulation can improve the acoustic insulation by 7 dB.
	Merli et al. (2018)	Monolithic, granular aerogel, and traditional air glazing systems	Compared to the traditional air-glazing system and the granular aerogel glazing, the monolithic aerogel glazing system can improve the sound insulation index by 3 dB, and by around 2 dB, respectively.
	Buratti et al. (2017)	Aerogel-based building materials	Compared to the absorption coefficient at 0.1 of conventional plasters, the coefficient was 0.29 for aerogel-based plaster.
Economic performance	Mujeebu et al. (2016)	Nano aerogel glazing	The nanogel glazing can reduce the energy consumption by 14%. Furthermore, the nanogel glazed windows with polystyrene foam in the wall and roof show the lowest system payback time at 7 years.
Others			
Hygrothermal behaviors	Nosrati and Berardi (2018)	Aerogel-enhanced insulating materials	Relative humidity has a more obvious effect on thermal conductivity than the temperature
long term aging	Berardi and Nosrati (2018)	Aerogel-enhanced insulating materials	The 20-year operation will increase the thermal conductivity by 10%.

The investigated variables and systems include granular and monolithic aerogel glazing systems in different orientations.

#### Thermal performances—transmitted heat gain, total heat gain, and heat gain

From the perspective of thermal and energy performances, depending on the application regions, the performance criteria include heat loss (in heating-dominated regions), transmitted heat gain, and total heat gain (in cooling-dominated regions). In subtropical regions, aerogel glazing systems have been studied, with respect to stochastic uncertainty analysis and optimal designs. The stochastic uncertainty can reduce the heat gain (Zhou and Zheng, 2020c). For the optimal geometrical and thermo-physical designs, compared to particle swarm optimization, the teaching-learning-based optimization can reduce the weekly total heat gain by 7.2% (from 6.9 to 6.4 kWh/m^2^) (Zheng and Zhou, 2019). Furthermore, the biobjective optimizations can reduce the heat gain to 322.4 kWh/m^2^ by 3.4% (Zhou and Zheng, 2020a).

#### Visual performances—haze impact and indoor illuminance

**Figure 6:**
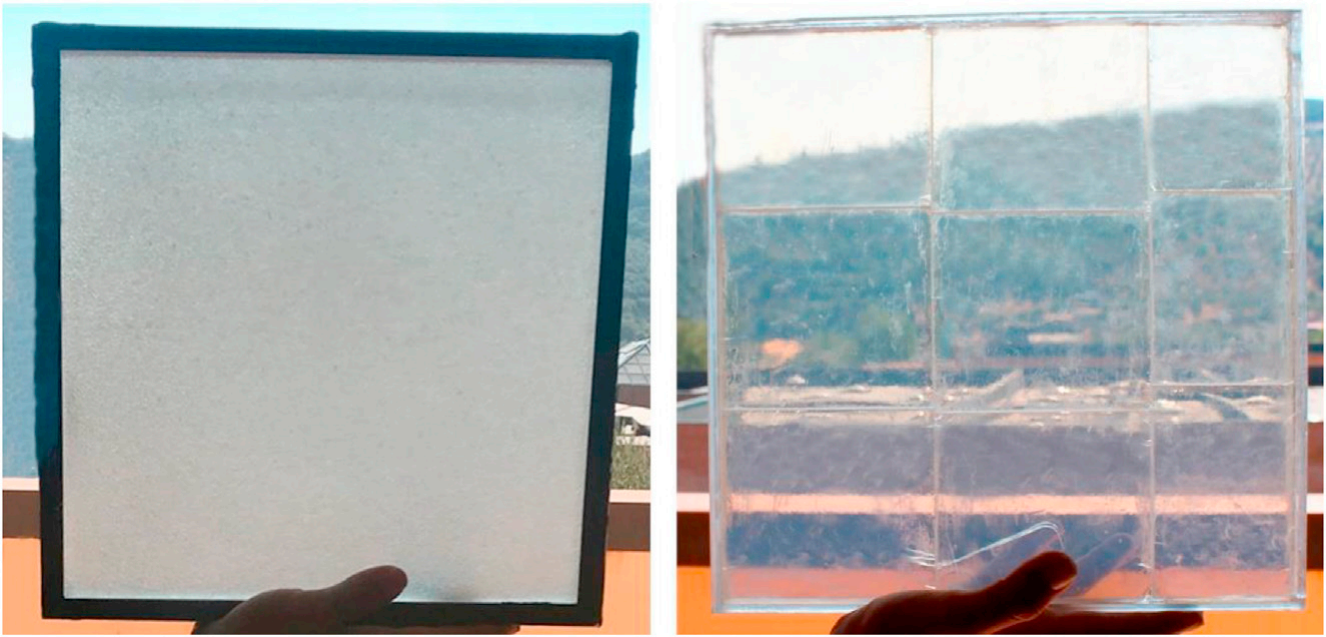
Aerogel glazing: granular (left side) and monolithic (right side) The light transmission of the monolithic silica aerogel glazing is 10% higher than a granular aerogel glazing, but 13% lower than conventional low-e double glazing. Figure 6 is reprinted from, Solar Energy, Zinzi et al., (2019). Optical and visual experimental characterization of a glazing system with monolithic silica aerogel. Copyright183, 30–39, with permission from Elsevier.

The visual performance of aerogel glazing systems has also been studied. Compared to the double-glazing, aerogel windows show a lower daylight transmission coefficient, owing to the opacity of aerogel particles (Garnier et al., 2015). Figure 6 shows the visual performance of granular and monolithic aerogel glazing systems (Zinzi et al., 2019). The light transmission of the monolithic silica aerogel glazing is 10% higher than a granular aerogel glazing, but 13% lower than a conventional low-e double glazing. The exploration of advanced aerogel materials has also been studied to improve the optical performance. It is noteworthy that the impact of aerogels on the indoor illuminance is dependent on the weather condition (Cotana et al., 2014).

Furthermore, for real applications, the visual performance of aerogel glazing systems is also dependent on the thermal performance. The comprehensive study of thermal and visual performances has also been studied. The biobjective optimizations on total heat gain and indoor illuminance (Zhou and Zheng, 2020a) can reduce the heat gain by 3.4%.

Furthermore, in the practical application, the haze will degrade the visual performance of optically transparent aerogels, restraining the application in windows. The impact of haze in aerogels applied to windows on the thermal and optical performances needs to be studied. Ideally, the aerogel glazing shows the most excellent optical performance when the haze is zero. Researchers focused on the haze prediction in aerogels and effective solutions to reduce the haze in transparent aerogels. In order to accurately predict the haze, a radiative transfer model was developed based on physical properties (Zhao et al., 2019). The model can help to develop low-haze silica aerogels through micro-structure design. Solutions have also been investigated to reduce diffuse transmittance and haze. The haze from the bulk in 0.017-g/cm^3^ aerogels was owing to Rayleigh scattering with bluish haze, and the opacity of 0.006-g/cm^3^ aerogels was owing to the Mie scattering (Mandal et al., 2019). Strategies need to be explored to keep the particle size low (Wang et al., 2020), such as increasing the rate of gelation. Polymers with 25 mol % pyromellitic dianhydride and 4,4′-hexafluoroisopropylidene di (phthalic anhydride) were successfully applied in the backbone structure, to produce polyimide aerogels with high optical transmission and low haze (Vivod et al., 2020).

#### Acoustic performance

Compared to traditional double-glazing systems, the acoustic insulation level is much higher for the aerogel glazing systems. For the aerogel glazing in the south façade, the aerogel can increase the acoustic insulation level from 28 to 31 dB (Cotana et al., 2014). Compared to the traditional air-glazing system and the granular aerogel glazing, the monolithic aerogel glazing system can improve the sound insulation index by 3 dB, and by around 2 dB (Merli et al., 2018), respectively.

In addition to the aerogel glazing system, aerogels have also been integrated into walls to improve the acoustic insulation level. The aerogel can improve the acoustic insulation level by 7 dB (Ibrahim et al., 2019). Compared to the absorption coefficient at 0.1 of conventional plasters, the absorption coefficient was improved to 0.29 (Buratti et al., 2017).

#### Economic performances

With the adoption of aerogel materials in building glazing systems, the operational cost saving can be realized owing to the energy savings, whereas the initial economic investment is increased. Economic feasibility of the aerogel glazing systems has also been studied. Furthermore, the nanogel glazed windows with polystyrene foam in the wall and roof show the lowest system payback time at 7 years. In an office building, the economic feasibility between nanogel aerogel glazing and PCM glazing systems was comparatively studied (Abuhenidy et al., 2019). Research results indicated that the most cost-effective solution is the nano aerogel glazing, owing to the considerable amount of annual energy savings by 11.71%, whereas the PCM glazing only reduces the annual energy consumption by 10.86%. The system payback time of the aerogel glazing is 4.4 years (Gao et al., 2016a, 2016b).

It is noteworthy that limited studies on the economic feasibility assessment were conducted for aerogel glazing systems. This is because under the current commercial market, the manufacture costs of the granular and monolithic aerogel glazing systems are quite high, resulting in the economic infeasible. Advanced technologies for aerogel glazing systems with low costs are highly desirable to improve the economic feasibility. Future studies can focus on the multi-dimensional economic performance assessment, including the levelized cost of energy, the discounted payback time, the profitability index, and the net present value.

### Outlooks for future modeling and system criteria

Table 6 summarizes performance comparison between physics-based models and ML models for aerogel glazing systems, with respect to different climates, material types, model prediction accuracy, running time, and economic performance. Based on the above-mentioned literature reviews, several challenges can be identified, with respect to advanced modeling development and multi-criteria performances improvement. In order to realize accurate performance predictions, future studies can be focused on the following topics:

1) development of resistance capacitance models to simplify the multi-criteria performances predictions (such as the thermal, visual, and acoustic performances);
2) development of advanced learning algorithms, optimal structural configuration, and learning epochs for accurate predictions on multi-criteria performances;

**Table 6:** Performance comparison between physics-based models and ML models for aerogel glazing systems in different climates

Model types	Studies	Climate	Material type	Variables	Approach	Performance	Model prediction accuracy and comparison	Results
Physics-based models	Zheng and Zhou (2019)	Hot summer and cold winter region, Changsha, China	Aerogel granule	Thermal conductivity, thickness of aerogel layer, extinction coefficient, density, specific heat capacity, and orientation angle of the glazing	Optical Model following interface energy balance method, and heat Transfer Modeling	Temperature	The root-mean-square error of the outer glass, aerogel, and inner glass are 1.1, 1.6, and 1. The relative error is less than 10%.	Good agreement between experimental and numerical results
	Li et al. (2020b)	Cold climate in China	PCMs-aerogel	Thermal conductivity, density, specific heat, and thickness	Radiative and convective heat transfer models	Temperature and heat flow on the inner surface	The average relative difference between the simulated and experimental surface temperature is 4.3%.	Silica aerogel insulation can effectively exploit latent heat of PCM, and the optimum silica aerogel thickness is 20–30 mm.
	Liu et al., (2019)	Hot summer and cold winter region, Changsha, China	Aerogel granules layer	Clear and overcast sky	Interface energy balance principle	Solar extinction coefficient	–	Solar extinction coefficient of nano-porous silica aerogel is 0.0392/mm and 0.0296/mm for the clear and overcast sky, respectively.
	Gao et al. (2014)	–	Aerogel granule	Particle size	Experimental testing in the lab	Heat loss, light transmittance	–	Compared to double glazing systems, aerogel glazing with particle size at 3–5 mm can reduce the heat loss by 58% and light transmittance by 38%. Furthermore, aerogel glazing with a particle size lower than 0.5 mm can reduce the heat loss by 63% and light transmittance by 81%.
	Gao et al. (2016a, 2016b)	A cold climate (Oslo, Norway)	Aerogel glazings	Particle size	Energy Plus	Energy saving, economic performance, and environmental impact	–	Compared to double glazing, aerogel glazing shows a 21% reduction in energy consumption. The return time on economic investment is 4.4 years
Mathematical fitting models	Zheng and Zhou (2019)	Hot summer and cold winter region, Changsha, China	Aerogel granule	Thermal conductivity, thickness of aerogel layer, extinction coefficient, density, specific heat capacity, and orientation angle of the glazing	Curve fitting method	Total heat gain	Coefficient of determination (R2) are 0.9405, 0.9511, 0.9609, 0.9697, 0.976 and 0.9768, for linear fitting-stepwise regression, linear fitting-lsqurvefit estimation, lsqurvefit estimation with exponent at 2, 3, 4 and 5, respectively.	The prediction accuracy of the mathematical fitting method is highly dependent on the mathematical function form.
Machine learning models	Zheng and Zhou (2019)	Hot summer and cold winter region, Changsha, China	Aerogel granule		Cross-entropy function	Total heat gain	Coefficient of determination (R^2^) at 0.9985 and 0.9993 when raining times are 5000 and 30,000, respectively.	The optimal solution through the teaching-learning-based optimization algorithm shows the total heat gain of 6.4 kWh/m^2^, 7.2% lower than that from the particle swarm optimization algorithm at 6.9 kWh/m^2^.
	Zhou and Zheng (2020a)	Subtropical region, Hong Kong	Aerogel granule	Thermal conductivity, thickness of aerogel layer, extinction coefficient, density, specific heat capacity, and orientation angle of the glazing	Machine-learning-based multi-objective optimization	Heat flux, total heat gain, and indoor illuminance	The time-duration was reduced from 1440 h to 12 h for the multi-objective optimization.	The multi-objective optimization can reduce the annual total heat gain by 31.9% and improve the annual indoor illuminance by 67.2%.

Model types mainly include physics-based models, mathematical fitting models, and machine learning models. Physics-based models mainly include optical models following the interface energy balance method and heat transfer models. Mathematical fitting models can establish a straightforward relationship between multivariant variables and multi-criteria, following different curve fitting methods. Machine learning models are developed following cross-entropy function through multiple linear regression, support vector regression, and backpropagation neural network.

From the perspective of multi-criteria performance assessment, several research topics are worthy to be well investigated:

1) the accurate performance predictions of the aerogel glazing and aerogel integrated systems, with uncertainty of the thermo-physical properties;
2) thermal and optical performances degradation over long-time operations, together with effective solutions for thermo-physical performance improvement, such as the vacuum sealing of aerogel layer to avoid oxidation and humidification;
3) the high-manufacture costs of the granular and monolithic aerogel glazing systems propose challenges for large-scale production and market acceptance. The development of advanced manufacture technologies is quite necessary to significantly reduce the cost of aerogels integrated components, and to improve the social acceptance and popularity in the commercial market;
4) with the adoption of aerogel glazing systems, the improvement in thermal insulating performance will result in a decrease in visuality. Thereafter, additional electricity consumption will be required through the lighting system to guarantee the minimum level of indoor illuminance. Optimal solutions are necessary to make the trade-off between thermal insulation and visual performance.

## Parametric analysis, single-, and multi-objective optimizations under deterministic scenario and stochastic uncertainty

The current literature mainly focuses on performance prediction of aerogel glazing systems under deterministic scenarios, such as deterministic weather profiles, constant thermo-physical properties, pre-scheduled indoor occupancy profile, and so on, whereas there are quite a few studies exploring system performance under stochastic uncertainties of input parameters. During the real application process, multivariants are with high-level uncertainties. The consideration of the high-level stochastic uncertainties is quite necessary, especially for the improvement of system reliability, robustness, and resilience. In this section, a comprehensive literature review has been conducted on deterministic and stochastic scenarios.

### Deterministic-based multi-criteria performance

In academia, studies are limited to single- and multi-objective optimizations to guide the optimal design on multiple input variables. The investigated variables include the thermo-physical parameters (such as the particle size of aerogel granules (Gao et al., 2014)), thermal conductivity, extinction coefficient, specific heat capacity (Zheng and Zhou, 2019), geometrical parameters, and the orientation (Chen et al., 2018). The system performance criteria include heat flux (Zheng and Zhou, 2019), total heat gain (Chen et al., 2018), heat loss (Chen et al., 2018), and light transmittance (Gao et al., 2014).

#### Parametric and sensitivity analyses

Parametrical analysis on geometrical and thermo-physical parameters has been studied, in recent years. The parametrical analysis was conducted on thickness (Cuce et al., 2014a, 2014b). For the cost minimization, the parametrical analysis on the thickness was conducted in different climate zones (Ibrahim et al., 2015). Results showed that the thickness between 1.7 and 4.4 cm can result in a relatively shorter payback period between 1.4 and 2.7 years. The effect of aerogel glazing orientation on total heat gain/total heat loss was quantitatively analyzed (Chen et al., 2018), and the most optimal orientations were identified as the south and the north. With respect to the particle size of aerogel granules, parametrical analysis on particle size was conducted (Gao et al., 2014), in terms of heat loss and light transmittance performance. Results showed that compared to conventional double-glazing systems, the aerogel glazing with particle size lower than 0.5 mm can reduce the heat loss by 63%.

Furthermore, with respect to the heat flux and total heat gain, sensitivity analysis on geometrical and thermo-physical parameters of aerogel glazing systems was conducted (Zheng and Zhou, 2019). The sensitivity results indicated that the total heat gain is mainly dependent on the orientation angle, whereas the heat flux is dominated by the extinction coefficient. The following-up research in different climates (Zhou and Zheng, 2020b) indicated that the influence order of each parameter on each objective also relies on local meteorological parameters.

#### Single- and multi-objective optimizations

Geometrical and operating parameters in the aerogel glazing mainly include orientation, extinction coefficient, density, specific capacity, thermal conductivity, thickness of the aerogel layer, and so on. The general principle for machine learning-based optimization can be summarized as follows:

1) develop the performance prediction model to establish the mathematical association between multiple variables and the multi-criteria (such as heat flux, total heat gain, illuminance, sound performance);
2) integrate the prediction model with optimization algorithms (such as non-dominated sorting genetic algorithm, teaching-learning-based optimization algorithm, particle swarm optimization algorithm, and so on);
3) search for the optimal solution following the principle of the selected optimization algorithm;
4) check the effectiveness of the optimal solution by running the original model for multi-criteria performance prediction.

Previous studies are mainly on the aerogel thickness for the single-objective optimization, whereas the consideration of conflicting multi-criteria with effective trade-off strategies is of great significance. It is noteworthy that the dimensional increase of optimized multi-variables will significantly increase the computational load for the search of the global optimal solution, as the optimization engine should iteratively return back to the physical model time by time. In order to improve the optimization efficiency, an artificial neural network-based optimization method was proposed (Zheng and Zhou, 2019), with the mechanism to replace the original physic-based model. Research results indicate that compared to particle swarm optimization, the teaching-learning-based optimization can reduce the weekly total heat gain from 6.9 to 6.4 kWh/m^2^ by 7.2%.

In addition to single-objective, multi-objectives include total heat gain (Zheng and Zhou, 2019), CO_2_ emissions (Guinoa et al., 2017), energy cost (Ibrahim et al., 2015), and so on. Thermal, lighting, and acoustic performances of silica aerogel glazing were studied (Cotana et al., 2014). The multi-criteria performance analysis results indicate that the aerogel can reduce the heating energy consumption by 50% and improve the façade acoustic insulation index by 3 dB. Considering the contradiction of each objective, a novel multi-objective optimization method was developed (Zhou and Zheng, 2020a). The biobjective optimizations can reduce the annual total heat gain to 322.4 kWh/m^2^ by 3.4%, but increase the annual indoor illuminance to 173 klux by 6.6%.

#### Research limitations and future prospects

It is noteworthy that most studies are on the parametric analysis, whereas quite limited studies are on the multi-variable optimizations, following the advanced and heuristic optimization algorithms (such as genetic algorithm, particle swarm optimization, heuristic teaching-learning-based optimization algorithm, and so on). The implementation of advanced optimization algorithms can help to search for more superior solutions, for system performance improvement. However, the optimal solution also relies on the adopted optimization algorithm. Research topics as listed later in discussion need to be investigated in following-up studies.

1) development of advanced algorithms or improvement on already developed algorithms to search for the most optimal solutions;
2) development of surrogate models with accurate prediction performances (such as thermal, energy, visual, and acoustic performances) to accelerate the optimization process;
3) along the Pareto optimal front, methodologies need to be proposed to identify the “best of the best” solution, and to guide system designers to select the trade-off solution between multi-objectives.

### Stochastic uncertainty-based multi-criteria performance

The current literature is mainly focused on deterministic parameters-based performance analysis, whereas the progress on stochastic uncertainty-based performances is quite slow. As a matter of fact, scenario parameters are full of high-level uncertainties. The multi-level performance analysis with considerations on stochastic uncertainty is quite necessary.

In academia, parametric and sensitivity analyses on stochastic uncertainty of scenario parameters have been conducted on heat flux and heat gain (Zhou and Zheng, 2020c). However, the main challenge is the accurate uncertainty quantification of multiple scenario parameters. To quantify the scenario uncertainties for different types, a Markov Chain Monte Carlo was adopted (Zhou and Zheng, 2020c). Results showed that the consideration of scenario uncertainty in Guangzhou will reduce the heat flux and heat gain.

In addition to the uncertainty analysis, a generic methodology for the uncertainty-based optimization on aerogel glazing systems (Zhou and Zheng, 2020d) was proposed in a subtropical region, Guangzhou. The underlying mechanism is to first prepare the stochastic cases with the quantified stochastic uncertainty, and then to train the optimization function, which can thereafter be implemented in advanced optimization algorithms to search for the optimal design parameters, avoid local optimization, and the overestimation. Compared with the deterministic scenario, the uncertainty-based optimization (Zhou and Zheng, 2020d) can further reduce the heat flux and heat gain.

**Figure 7:**
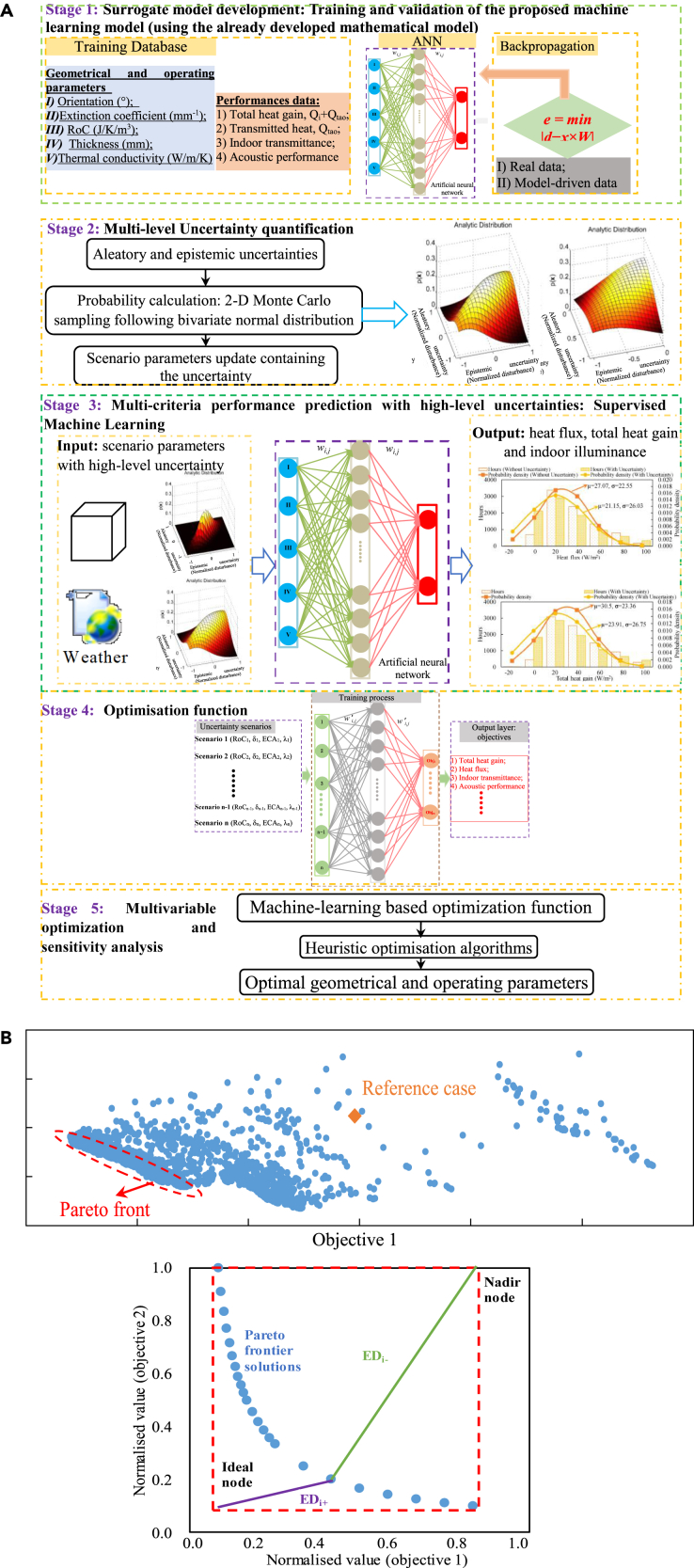
The roadmap for uncertainty-based multi-objective optimization (A) the optimization process. The stochastic uncertainty-based multi-objective optimization includes: (1) accurate surrogate models for multi-criteria performance prediction, (2) uncertainty quantifications of scenario parameters, (3) stochastic uncertainty-based performance predictions, (4) multi-objective optimization function in accordance with performance under stochastic uncertainty, (5) the search for optimal solutions through heuristic optimization algorithms. (B) The post-multi-criteria decision-making process (Zhou et al., 2020e). The post-multi-criteria decision-making process is to identify the “best of the best” solution, along the optimal Pareto front. The current literature provides several multi-criteria decision-marking methods, including the Shannon entropy (Falsafi et al., 2014), Euclidean distance-based methods (such as the LINMAP (Feng et al., 2015a, 2015b), and TOPSIS (Yousefi et al., 2017) decision approach), fuzzy membership function method (Parreiras et al., 2006; Yuan et al., 2017), and the evidential reasoning method (Li et al., 2018). However, no one method always performs the best (Jing et al., 2019). The selection of the “best of the best” solution is dependent on several critical factors, such as the prioritized objective, the benefits of different stakeholders, and so on. Researchers are suggested to focus on effective methodologies to determine the “best of the best” solution. Figure 7 is partially reprinted from, Renewable and Sustainable Energy Reviews, Zhou et al., (2020e). Passive and active phase-change materials integrated building energy systems with advanced machine-learning-based climate-adaptive designs, intelligent operations, uncertainty-based analysis, and optimizations: A state-of-the-art review. Copyright 130, 109,889, with permission from Elsevier.

Figure 7 shows the roadmap for uncertainty-based multi-objective optimization, formulated by the author. As shown in Figure 7A, the stochastic uncertainty-based multi-objective optimization includes: (1) accurate surrogate models for multi-criteria performance prediction, (2) uncertainty quantifications of scenario parameters, (3) stochastic uncertainty-based performance predictions, (4) multi-objective optimization function in accordance with performance under stochastic uncertainty, and (5) the search for optimal solutions through heuristic optimization algorithms. Figure 7B shows the post-multi-criteria decision-making process to identify the “best of the best” solution, along the optimal Pareto front. The current literature provides several multi-criteria decision-marking methods, including Shannon entropy (Falsafi et al., 2014), Euclidean distance-based methods (such as the LINMAP (Feng et al., 2015a; 2015b), and TOPSIS (Yousefi et al., 2017) decision approach), fuzzy membership function method (Parreiras et al., 2006; Yuan et al., 2017), and evidential reasoning method (Li et al., 2018). However, no one method always performs the best (Jing et al., 2019). The selection of the “best of the best” solution is dependent on several critical factors, such as the prioritized objective, the benefits of different stakeholders, and so on. Researchers are suggested to focus on effective methodologies to determine the “best of the best” solution.

### Discussion on future prospects

With respect to the single- and multi-objective optimizations under deterministic and stochastic uncertainty, technical challenges are clarified as shown later in discussion to serve as avenues for the following-up research.

1) Most researchers focused on parametrical analysis and single-objective optimization based on deterministic parameters. The multi-objective optimization of aerogel glazing systems is limited under high-level scenario uncertainties. Future studies are expected to cover the scientific gaps during the realistic operation process.
2) Compared to the single-dimensional deterministic scenario, the multi-dimensional uncertainty scenarios will propose challenges on performance prediction, mainly owing to various combinations of stochastic parameters. Effective tools with straightforward input–output schemes need to be developed for efficient performance prediction without sacrificing the accuracy.
3) Development of efficient optimization engines for stochastic uncertainty-based optimizations. Trade-off solutions need to be explored between the robustness of optimal results and computational efficiency throughout the whole optimization process.
4) During the post-multi-objective optimization process, the development of methodology for the multi-criteria decision making (MCDM) based on stochastic uncertainty scenarios is critical to assist system designers to make the most appropriate decision along the Pareto optimal front.

## Limitations of study, outlook, and recommendations

### Advanced composite aerogel materials and novel PCMs integrated aerogel glazing systems

Aerogel materials show promising prospects in building energy savings, owing to super-insulation property, sound-proof characteristic, and translucent property for visibility. Furthermore, the integration of thermal-insulated aerogel with PCMs with latent heat storage will increase the solar energy utilization efficiency and reduce thermal loss to the surrounding environment. However, considering scientific gaps in academia, future studies can be focused on the following topics:

1) advanced composite aerogel materials with high visibility, cost competitiveness, social acceptance, and widespread popularity in the commercial market;
2) optimal structural designs on aerogels integrated systems, together with smart system controls, to improve the robustness, reliability, and resilience under extreme conditions. Furthermore, frontier guidelines need to be provided for aerogel glazing applications in different climates.
3) integrated solutions need to be explored for accurate predictions in multi-criteria performances under boundary condition change and degradation on thermophysical properties, such as resistance–capacitance (RC) models and human knowledge-based machine learning.

### Stochastic uncertainty-based optimizations

Optimizations on aerogel glazing systems during design and operation processes can improve multi-criteria performances (such as thermal, acoustic, and visual performances), increase economic competitiveness with optimal geometrical design parameters, and provide concrete guidelines for reliable system operations. Furthermore, the consideration of stochastic uncertainty of scenario parameters can avoid the overestimation or underestimation of aerogel systems in realistic operations. For the single- and multi-objective optimizations on aerogel glazings under deterministic scenario and stochastic uncertainty, following-up studies can focus on:

1) model development for accurate uncertainty quantifications of thermo-physical parameters, such as thermal conductivity, extinction coefficient, specific heat capacity, and so on. Combined solutions with diversified probability density functions will be effective solutions to characterize multi-diversified stochastic uncertainties;
2) effective prediction tools with high efficiency and accuracy are desirable for multi-criteria performance predictions with combinations of stochastic parameters, such as human knowledge-based data-driven models, machine learning-based surrogate models, and so on;
3) stochastic uncertainty-based optimizations need to be studied, to improve the reliability, and robustness. The ultimate objective for stochastic uncertainty-based optimizations is to find the global optimal solution, which shows more reliable performances than any stochastic scenario;
4) after the multi-objective optimizations, the identification of the “best of the best” solution along the Pareto front is worthy to be investigated, especially comparing results driven by different multi-criteria decision making (MCDM) approaches. Regarding the priority sequences of each objective within system designers, the on-site questionnaire is a straightforward and effective solution to allocate the priority coefficient to each objective.

### Challenges and outlook for future trends

With respect to aerogel glazing applications in buildings with components prefabrication, modeling development, single-, and multi-objective optimizations under deterministic and stochastic uncertainty, several technical challenges, and potential prospects are summarized later in discussion.

1) The fragility of monolithic aerogel leads to a technical difficulty in production. High manufacturing costs of the granular and monolithic aerogel glazing systems propose challenges on the widespread market acceptance. Furthermore, degradation of aerogel materials will propose challenges for long-term stability operation. Researchers can focus on advanced aerogel materials with high strength during the production process, by controlling surrounding, and working conditions for production (such as pH, concentration, water content, and so on);
2) Transparent aerogel plays significant roles in solar energy utilization, owing to the high thermal insulation performance. Meanwhile, complementary function between aerogels and energy storage materials (such as PCMs) can comprehensively improve the overall performance of the glazing system. Future studies can focus on novel structural designs and operational controls on the aerogel material integrated glazing systems;
3) Machine learning techniques with advanced algorithms can provide innovative approaches to address the complexity, inaccuracy, and inefficiency for performance prediction of aerogel glazing systems. However, in order to guarantee the performance prediction accuracy of the well-trained surrogate model via machine learning, a large amount of the database needs to be prepared. In order to address this issue, human knowledge-based machine learning can reduce abundant data requirement, increase performance prediction reliability, and improve interpretability for non-professionals or multidisciplinary research (Deng et al., 2020). Furthermore, in order to address the computational complexity owing to the convergence of iterative calculation, when conducting parametrical and comparative analysis, single-, and multi-objective optimization, the database can also be prepared and generated from physics-based models, reducing the experimental and labor cost;
4) In order to improve the prediction performances under uncertainty, machine learning techniques can train the surrogate models, whereas the prediction performance highly relies on the adopted training algorithms and the structural configuration of the neural work. Future studies can focus on the applicability of different training algorithms, total training epoch, and structural configurations, for accurate predictions on multi-criteria performances (such as heat flux, transmitted heat gain, total heat gain, light transmittance, and acoustic performance);
5) Advanced optimization methodology needs to be developed to design the geometrical, thermo-physical, and operating parameters of aerogel glazing systems.

## Conclusions

In this study, a holistic review was conducted on aerogel glazing applications in buildings: components prefabrication, modeling development, single- and multi-objective optimizations under deterministic and stochastic uncertainty. Contributions of this study include an innovative modeling approach for accurate thermal/visual performance prediction under coupling effect of complex structures in aerogels, straightforward mathematical association between multivariant and multi-objective with competitive computational efficiency, user-friendly interface for non-professionals or multidisciplinary research works, together with robust design for stochastic uncertainty and uncertainty-based optimization. An in-depth analysis has been provided, on the aerogel material production, component prefabrication, and building applications. Owing to the complexity of nanoparticle structure in aerogel materials and degradation over long-term operation, critical parameters with high-level uncertainties for heat and light transmissions mainly include extinction coefficient and thermal conductivity for heat transfer in porous aerogels. Adopted methodologies for thermal/visual performance prediction and uncertainty quantification mainly include interface energy balance, Rosseland approximation, and Monte Carlo method. Furthermore, in order to promote the reliable system design, interdisciplinary machine learning has been applied for parametric analysis, parameter optimizations under deterministic scenario, and stochastic uncertainty. The formulated novel structural designs, data-driven model, stochastic uncertainty-based analysis, and optimizations can promote aerogel materials in energy-efficient buildings. Key conclusions are listed later in discussion:

1) In the academia, main sources for aerogel materials production include industrial biowastes and used materials recycling. The structural strength of the silica network is dependent on pH value, concentration, and water content. The novel combined aerogel glazing system designs, such as PCM-aerogel glazing systems, are full of prospects, through synergistic functions between aerogel with high thermal insulation and PCMs with latent heat density;
2) Modeling development for multi-criteria predictions is systematically reviewed, including mathematical and data-driven models. Technical challenges for accurate performance predictions include uncertainty quantification of thermo-physical properties owing to aleatory/epistemic uncertainty and degradation over the long-term operation, multi-dimensional predictions with stochastic scenario uncertainties, development of advanced learning algorithms, optimal structural configuration, and learning epochs for accurate predictions on multi-criteria performances;
3) Previous studies are mainly on parametric analysis and single-objective optimization based on deterministic parameters, whereas there is limited progress on multi-objective optimizations under high-level scenario uncertainties. Compared to the single-dimensional deterministic scenario, the multi-dimensional uncertainty scenarios will propose challenges on performance predictions, mainly owing to various combinations of stochastic parameters. The interdisciplinary application of machine learning in the development of multi-criteria surrogate models can be an effective solution for efficient and accurate predictions. Furthermore, the development of efficient optimization engines for stochastic uncertainty-based optimizations is quite necessary for reliable and robust designs. Trade-off solutions need to be explored between the robustness of the optimal results and the computational efficiency throughout the whole optimization process.
4) Machine learning techniques for surrogate model development require a large amount of database with considerable labor and experimental costs. Integrated solutions need to be explored for accurate predictions in multi-criteria performances under boundary condition change and degradation on thermophysical properties, such as resistance–capacitance (RC) models and human knowledge-based machine learning. Furthermore, human knowledge-based machine learning can reduce abundant data requirement, increase performance prediction reliability, and improve interpretability for non-professionals or multidisciplinary research.

Through the comprehensive literature review, aerogel materials are promising candidates for energy-efficient buildings. In order to promote the aerogel materials' applications in buildings, combined efforts need to be paid to the advanced composite aerogel materials and novel PCMs integrated aerogel glazing systems, stochastic uncertainty-based single- and multi-objective optimizations, and economically competitive production of aerogel materials for the large-scale production and widespread market acceptance.

## Nomenclature

Symbols
  e Blackbody flux
  *k* Effective radiative conductivity
  K Rosseland mean extinction coefficient
  n Effective refractive index
  T Temperature
  λ Wavelength
  σ Stefan–Boltzmann constant
Acronyms
  MCDM Multi-criteria decision making
  PCCA Phase-change composited aerogels
  PCM Phase-change material
  RC Resistance–capacitance
  SAC Sound absorption coefficient
